# Adaptation of a mobile phone health survey for risk factors for noncommunicable diseases in Colombia: a qualitative study

**DOI:** 10.1080/16549716.2020.1809841

**Published:** 2020-08-28

**Authors:** Angelica Torres-Quintero, Angela Vega, Dustin G. Gibson, Mariana Rodriguez-Patarroyo, Stephanie Puerto, George W. Pariyo, Joseph Ali, Adnan A. Hyder, Alain Labrique, Hannah Selig, Rolando Enrique Peñaloza, Andres I. Vecino-Ortiz

**Affiliations:** a Institute of Public Health, Pontificia Universidad Javeriana, Bogotá, Colombia; b Department of International Health, Johns Hopkins Bloomberg School of Public Health, Baltimore, MD, USA; c Institute of Bioethics, Pontificia Universidad Javeriana, Bogotá, Colombia; d Department of Global Health, Milken Institute School of Public Health, George Washington University, Washington, DC, USA

**Keywords:** Mobile phone surveys, noncommunicable diseases, risk factors, digital health, mHealth, health surveys, low- and middle-income countries

## Abstract

**Background:**

Data collection on noncommunicable disease (NCD) behavioral risk factors has traditionally been carried out through face-to-face surveys. However, its high costs and logistical difficulties can lead to lack of timely statistics for planning, particularly in low and middle-income countries. Mobile phone surveys (MPS) have the potential to fill these gaps.

**Objective:**

This study explores perceptions, feasibility and strategies to increase the acceptability and response rate of health surveys administered through MPS using interactive voice response in Colombia.

**Method:**

A sequential multimodal exploratory design was used. We conducted key informant interviews (KII) with stakeholders from government and academia; focus group discussions (FGDs) and user-group tests (UGTs) with young adults and elderly people living in rural and urban settings (men and women). The KII and FGDs explored perceptions of using mobile phones for NCD surveys. In the UGTs, participants were administered an IVR survey, and they provided feedback on its usability and potential improvement.

**Results:**

Between February and November 2017, we conducted 7 KII, 6 FGDs (n = 54) and 4 UGTs (n = 34). Most participants consider MPS is a novel way to explore risk factors in NCDs. They also recognize challenges for their implementation including security issues, technological literacy and telecommunications coverage, especially in rural areas. It was recommended to promote the survey using mass media before its deployment and stressing its objectives, responsible institution and data privacy safeguards. The preferences in the survey administration relate to factors such as skills in the use of mobile phones, age, availability of time and educational level. The participants recommend questionnaires shorter than 10 minutes.

**Conclusions:**

The possibility of obtaining data through MPS at a population level represents an opportunity to improve the availability of risk-factor data. Steps towards increasing the acceptability and overcoming technological and methodological challenges need to be taken.

## Background

Traditionally, data collection for public health purposes has been carried out in the context of health surveys through in-person interviews. However, these surveys take time and are costly, which makes it difficult for policy-makers to have up-to-date monitoring data on the prevalence of risk factors for non-communicable diseases (NCD) and other conditions, in particular in low- and middle-income countries (LMICs) with isolated populations distributed across broad geographic areas.

For these reasons, alternative strategies to enhance the timeliness and reduce the cost of health surveys are needed. One potential solution is to use mobile phone technology, which could reduce implementation and personnel costs and has the potential to provide reliable estimates given widespread coverage [1–3].

While high-income countries have long used telephone and mobile phone surveys to collect population health estimates, their use in LMICs is still fairly limited [4–6].

However, according to the World Health Organization’s (WHO) 2011 Mobile Health (mHealth) report, health surveys and surveillance were two of all the other mHealth initiatives with higher adaptation rates for participating Member States in the low and lower-middle income groups in comparison to how frequently the high- and upper-middle-income groups used mobile phones for data collection and surveillance. These were 40% for surveillance and 37% for health surveys in the low-income and 27% for surveillance in the lower-middle-income groups. The WHO reported that given the high penetration rate of mobile technology in LMICs (91.8% in 2015) that continues to increase, there exists a growing potential for the application of mHealth initiatives as a feasible route for countries with resource-constrained conditions and thus further research should be carried out focused on the viability and acceptability of mHealth initiatives in LMICs [7]. Other research indicated the added value in assessing users’ perspectives of perceived opportunities and barriers and incorporating this feedback throughout the development stages of the mHealth initiative, yielding higher user acceptability towards this technology and its application [8].

Previous research has assessed the development of health surveys through interactive voice response (IVR), where participants select responses to prerecorded questions via keypad or voice recognition software [9] and short message service (SMS; text message) technology to determine effectiveness for surveillance of NCD [4,10,11]. These surveys constitute a potential effective monitoring mechanism to assess NCD risk factors in the context of policy change, the implementation of programs, or the surveillance of secular trends [6,10,12]. This is possible since new methodological features have been developed to improve the population representativeness of the data and that its results can be comparable to face-to-face interviews in LMIC settings [13,14]. These tools can also be used to increase the opportunity for epidemiological surveillance and the collection of timely data, which is particularly useful in LMICs [8], where periodic population assessments are infrequent and expensive to carry out on a regular basis [15], leading to long periods without timely data [16].

In the context of the Bloomberg Philanthropies Data for Health Initiative (BD4HI) [17], research teams at the Pontificia Universidad Javeriana and the Johns Hopkins University are assessing the feasibility, validity and quality of the data collected through the deployment of mobile phone surveys (MPS) to monitor the prevalence of risk factors for NCDs [18] in Colombia.

In this study, we explored: 1) perceptions, attitudes and acceptability of answering and/or completing a survey on NCDs and associated behavioral risk factors via mobile phone surveys, 2) understanding of the language used in the questionnaire and preferred platform to answer a survey (IVR or SMS); and 3) possible challenges that might arise during the deployment of the surveys and ways to address them.

We analyze the perspectives from national experts, key informants and groups of potential users on the potential strategies to improve MPS completeness and accuracy. The findings can be used to help improve the quality and representativeness of MPS and therefore be useful in filling particular informational gaps related to the implementation of MPS to monitor population health.

## Methods

This paper presents the qualitative results of a larger study [19] that used a sequential multimodal exploratory design (QUAL → QUAN), in which an initial phase of qualitative data collection and analysis is developed, followed by another where quantitative data are collected and analyzed [20,21]. The purpose of this design is to adapt and test a standardized MPS instrument in the specific context where it will be applied and obtain generalizable and replicable results in subsequent studies [21]. In this study, three qualitative techniques were used: focus group discussions (FGDs), user-group tests (UGTs) and key informant (KI) interviews.

A search was performed for documents published between 2001 and 2019. The search involved peer-reviewed and gray literature using an integrated database that includes the following search engines: EBSCOhost, Clinical Key (Elsevier), Ovid, PubMed (incorporating Medline), EMBASE, JSTOR, ScienceDirect, Scopus, SpringerLink, ProQuest and Mendeley. The search consisted of the following terms used with AND/OR: mobile phone surveys/MPS, MPS in LMICs, non-communicable diseases/NCD, risk factors, mHealth, mHealth for NCDs in LMICs and health surveys.

The inclusion criteria for participation in FGDs and the UGTs were sex, age and rural or urban residence. Groups were formed by sex (men and women) age (18–25 years old, 26–65 years old and older than 65) and location (residents from Bogotá or nearby rural towns). The groups were set up according to these three criteria to account for preferences in the use of mobile phones, as well as possible differences in technological literacy (Table 1). The only exclusion criterion was being under 18 years of age. Groups were obtained through purposive sampling. They were approached through recruiters from the same community. All FGDs and UGTs were conducted in neutral areas close to the home or work location of the interviewees, only in the presence of participants and researchers.

**Table 1.** Inclusion criteria for focus group discussions (FGD) and user-group tests (UGT).

Inclusion Criteria	Focus Group Discussions						IVR User-group Testing			
	1	2	3	4	5	6	1	2	3	4
Female	X		X		X		X		X	
Male		X		X		X		X		X
Younger (18–25 years-old)	X			X			X	X		
Adults (26–65 years-old)		X	X		X				X	X
Older (66 and up)						X				
Urban Residency	X	X	X				X	X		
Rural Residency				X	X	X			X	X

FGDs and UGTs were conducted in Spanish by two or three Institute of Public Health researchers – a moderator (AT, a female interviewer with a master’s degree) and one or two note-taker/participant observer (also female team members, with master’s degrees or currently pursuing a master’s degree). Most of the participants of the FDGs and UGTs had no established previous relationships with the research team and did not know the researchers. The interviewer followed a pre-designed guide during the interview which lasted about two hours. The note-taker registered central aspects of the discussion and relevant elements of participants’ verbal and non-verbal expressions in field notes. Before beginning the FGD and UGT sessions, a brief questionnaire of 11 items was administered to collect basic demographic details from participants such as educational level, occupation and current ownership and use of a mobile phone.

For FGDs and UGTs, we translated a standardized semi-structured questionnaire from English into Spanish and back-translated to English. This semi-structured questionnaire was used in similar studies in Bangladesh, Tanzania and Uganda with adaptations made for each country. Each FGD and UGT included 7 to 10 people in order to facilitate participation and lasted about two hours. All FGDs and UGTs were led by the author (AT). FGDs were conducted to explore issues related to previous experiences responding to telephone surveys in general and specifically in health, availability and willingness to respond to surveys on NCD risk factors, preferences in the administration of the survey and in its duration, preferred profile of the interviewer and perception of incentives as a potential stimulus to increase response rates. UGT participants were sent an SMS or IVR survey to their personal mobile phone. The main purpose of this group was to allow participants to have a direct experience answering the survey and to provide feedback on it. Like the FGD, participants were asked to comment on potential challenges with administering the survey in Colombia and recommendations to improve its deployment.

Furthermore, we conducted semi-structured interviews, lasting up to an hour and a half, with key informants from Colombia to learn from their previous experiences in designing health surveys on similar topics and to obtain recommendations that could help improve the MPS. Key informants included researchers, government specialists and decision-makers, experts in design and implementation of public health policies and others experienced in the use of surveys of behavioral risk factors associated with NCD. The interviews, which were also conducted in Spanish, followed a semi-structured guide that emphasized research, policy and practice considerations related to MPS planning and implementation. A male member of the research team (AVO) conducted most of the interviews. Key informants were reached out through purposive sampling. They were approached directly by the interviewer. All interviews were conducted in the work location of the interviewees and only in the presence of the participant and researchers. The interviewer followed a semi-structured interview guide and the interview session lasted about one hour.

The narrative data obtained were transcribed and codified by two coders into *a priori* categories, as shown in Table 2 using NVivo. This facilitated the analysis and triangulation to obtain saturation levels needed to identify dominant tendencies in perception, preferences and recommendations on the survey’s application. Consensus and dissent were included as well to qualify and provide context for the results.

**Table 2.** A priori categories contemplated for the instrument and data analysis.

Category	Focus Groups	User Groups	Key Informant Interviews
Previous experiences as interviewees	X	X	X
Attitude towards the survey	X	X	X
Perceptions on quality of the information obtained on phone survey	X		X
Preferences on survey delivery method (IVR or SMS)	X	X	
Conditions for a successful phone survey	X		X
Incentives that motivate participants to finish the survey	X	X	X
Relevant information in the introduction and/or informed consent of the survey	X	X	
Recommendations on adjustments for the survey		X	

The institutional review boards of both the Johns Hopkins University Bloomberg School of Public Health and the Institute of Public Health of Universidad Javeriana approved this study. All participants provided written informed consent and permitted recording and use of information provided for research purposes. COREQ guidelines for reporting qualitative research were followed in this study [22].

## Results

Between February and November 2017 (Table 3), we conducted seven KI interviews, six FGDs (n = 54 participants) and four UGTs (n = 34 participants). The six FGDs consisted of male/urban (n = 2 FGDs), female/urban (n = 2), male/rural (n = 1) and female/rural participants (n = 1). With the exception of FGD #3, the FGDs were comprised of participants with a range of educational backgrounds and occupations.

**Table 3.** Focus group participants’ demographics.

Variables		FGD1		FGD2		FGD3		FGD4		FGD5		FGD6		Total	
		N	%	N	%	N	%	N	%	N	%	N	%	N	%
Sex															
	Women	11	100%			9	100%			10	100%			30	56%
	Men			10	100%			6	100%			8	100%	24	44%
Age (years)															
	18–25	11	100%					6	100%					17	31%
	26–29					2	22%			1	10%			3	6%
	30–39			1	10%					5	50%			6	11%
	40–49			7	70%					1	10%			8	15%
	50–59			2	20%	3	33%			1	10%			6	11%
	>59					4	44%			1	10%			5	9%
	Missing value									1	10%	8	100%	9	17%
Location															
	Rural							6	100%			8	100%	14	26%
	Urban	11	100%	10	100%	9	100%			10	100%			40	74%
Occupation															
	Domestic employee									1	10%			1	2%
	Student	11	100%					4	67%					15	28%
	Private company			10	100%					2	20%			12	22%
	Government employee							1	17%	1	10%	1	13%	3	6%
	Housekeeper					8	89%			5	50%			13	24%
	Self-employed					1	11%					3	38%	4	7%
	Farming									1	10%			1	2%
	Volunteer											4	50%	4	7%
	None							1	17%					1	2%
Higest education level															
	Elementary school			5	50%			4	67%	4	40%	8	100%	21	39%
	Middle school	6	55%	1	10%			1	17%		0%			8	15%
	High school	3	27%	2	20%					4	40%			9	17%
	Technical Professional			1	10%	5	56%			2	20%			8	15%
	Undergraduate	*2*	18%	*1*	10%	*4*	44%	1	17%					*8*	*15%*
	*Observations*	*11*		*10*		9		6		10		8		54	1

Table 4 shows a diversity in occupation and education level among participants of the UGTs. In terms of occupation, most of the people in UGT#1 worked as self-employed (71%) whereas most people in UGT #2 and #3 were mostly students and those in UGT #4 were housekeepers (57%). Meanwhile, 90% of the people in UGT #2 and #3 were students. Participants in UGTs had an educational level of high school (35%) and middle school (29%).

**Table 4.** User-group participants’ demographics.

		UGT1		UGT2		UGT3		UGT4		Total	
Variables		N	%	N	%	N	%	N	%	N	%
Sex											
	Women			10	100%			7	100%	17	50%
	Men	7	1			10	100%			17	50%
Age (years)											
	18–25			10	100%	10	100%			20	59%
	26–29							1	14%	1	3%
	30–39							2	29%	2	6%
	40–49	3	43%					3	43%	6	18%
	50–59	2	29%					1	14%	3	9%
	>59	2	29%							2	6%
Location											
	Rural	7	1					7	100%	14	41%
	Urban			10	100%	10	100%			20	59%
Occupation											
	Domestic employee							1	14%	1	3%
	Student			9	90%	9	90%			18	53%
	Private company	1	14%					2	29%	3	9%
	Housekeeper							4	57%	4	12%
	Self-employed	5	71%							5	15%
	Volunteer	1	14%							1	3%
	None			1	10%	1	10%			2	6%
Highest education level											
	Elementary school	2	29%					2	29%	4	12%
	Middle school	2	29%	6	60%			2	29%	10	29%
	High school			3	30%	7	70%	2	29%	12	35%
	Technical Professional	2	29%			1	10%			3	9%
	Undergraduate			*1*	10%	*2*	20%	1	14%	4	12%
	Postgraduate	*1*	14%							1	3%
*Observations*		*7*		*10*		10		7		34	

### Previous experiences and attitudes towards surveys

When questioned about their previous experiences answering surveys (whether on the phone or face-to-face), participants of all groups declared that financial companies, mobile phone operators, or health service providers had asked them to undergo similar surveys to explore their satisfaction levels with the services provided. Rural and urban adult women’s FGD and rural adult men’s UGT reported to have had participated in household surveys and population census.

Mostly, participants gave a negative appraisal of surveys of any kind (including face to face) because these studies are perceived as lengthy and inconvenient as they interfere with every-day activities and routines, and that factors might lead them to reject the survey or to answer them quickly or non-truthfully. Nonetheless, this was not a perception shared among the rural adult women FGD and rural senior men, who were said to have more time available to respond.

On the other hand, all participants showed apprehension to take calls from unknown numbers, especially for a phone survey. All groups alluded to how common it is in Colombia to use surveys for criminal purposes and deceiving people into giving up personal information in exchange for economic incentives. Adult men and women, both rural and urban, in the FGDs, and rural adult men during a UGT shared victimization experiences mentioning their intention to avoid and reject any such contacts.

It happened to my husband. He was allegedly called from a bank asking about how he had liked the service, that they were performing an evaluation, and they obviously weren’t from the bank because they started asking for credit card numbers and passcodes. That’s why I always hang up when I receive survey calls. (Urban adult women FGD)

### Survey introduction and trust

Several groups, especially the KI, provided recommendations that were thought to enhance trust in MPS. The suggestion that survey introductions include descriptions of: (a) how investigators gained access to the participant’s phone number; (b) the scope of the questionnaire, clarifying that financial or private information will not be requested and that the phone call is free of charge; and (c) to inform about the study’s purposes and how the data will be used to assist health authorities on the design of prevention programs for NCD.

Similarly, nearly all FGD groups suggested to include an email or web address to allow survey respondents to confirm which entity was conducting the survey and corroborate the information given at the beginning of the survey. The KIs recommended hiring a call-center to answer any concerns and complaints and provide additional information requested by the participants.

Additionally, many participants agreed on the fact that implementing a mass media campaign through local and national channels prior to the survey’s implementation will inform citizens and encourage them to answer. They also suggested to indicate the phone number from which the survey will be sent, to reduce rejections due to reluctance to answer a call from an unknown number.

I think that for people to answer this survey on the phone, a unique phone line should be established so one can know to expect a call from that specific number, and write it down. The number needs to be shown on TV and they need to say that one will be receiving the call so that one isn’t surprised by it. (Rural adult women FGD)

### Provision of incentives

The moderators asked the FGD and UGT participants about their perceptions of introducing a 2 USD equivalent incentive in the form of airtime for completing a survey that would last approximately 20 minutes. In this regard, most of the participants expressed disagreement, since the offer of prizes or money is usually a strategy commonly used by criminal groups to extort money from the population through mobile phone calls. Therefore, inclusion of information on incentives could reduce credibility and confidence in the study. Additionally, some of the participants pointed out that offering money to answer the survey could lead people to ‘answer whatever comes up to their mind’ to make quick cash, instead of giving true information about their health risk behaviors. Instead, KIs, FGD urban women and men (25 to 65 years old) and rural women (25 to 65 years old) suggested to change the economic incentive to a recognition of their social contribution. The younger urban women and adult urban men FGD and all of the UGTs suggested to provide the incentive at the end of the survey and to avoid giving information about incentives in the introduction to reduce the probability that this factor negatively interferes in the veracity of the answers or generate reluctance to participate associated with the aforementioned security concern.

After the survey application, the FGD participants stated that the incentive offered of the equivalent of 2 USD was unattractive for the following reasons: (a) they perceive an unbalanced relation between cost (time invested answering the survey) and benefit (amount received), namely because they perceived that the time invested was greater than the incentive received; (b) some of the participants have post-paid (i.e. unlimited airtime) call plans so it was not an advantage to receive additional minutes unless these were provided in form of credit, which is not always technologically possible; (c) mobile phone coverage in rural areas is not as good as in the cities and the lack of technological skills or other cultural aspects might discourage them from participating; and (d) urban participants use their mobile phones less frequently to make calls. Communication is carried out primarily through social networks, especially among young people. Participants proposed alternative incentives that suit them better on their particular needs and context. Adult women and men proposed health-related incentives such as information on NCDs, first aid kits or manuals, young women were more interested in transportation tickets or food bonuses, and finally, young men proposed incentives such as subscriptions for digital and videogame platforms.

### Additional preferences and conditions for successful phone survey implementation

FGD and the UGT participants suggest that the favorable attitude to answer a telephone survey about risk behaviors associated with NCDs is associated with factors such as implementation modality (face-to-face, IVR or SMS), survey duration, personal time availability, population profiles (health condition, socio-economic and educational level, occupation), security and confidentiality concerns, the nature of the questions and the intended use of data.

In this sense, as the first option, participants from all the groups agree they prefer face-to-face surveys; as the second option, the surveys carried out through the Call Center (CATI) and, the last option, considering them impersonal, the IVR surveys. They explained that direct contact with the operator allows them to solve doubts that might arise, repeat questions and provide truthful information since direct contact exerts social pressure that decreases the likelihood of ‘telling lies’. In contrast, a survey with pre-recorded messages was thought to encourage rejection by most participants. Participants from the rural senior men FGD showed difficulties related to their abilities and the handling of the devices that interfere in responding to the survey. Despite these limitations, KIs stated that telephone surveys can increase response rates in higher socio-economic groups, which are otherwise reluctant to answer face-to-face surveys.

Although in principle the modality of survey through SMS was perceived by the UGT and KI participants as a potentially more attractive format, due to the advantages of an asynchronous response and the possibility of reviewing the question in the text, the participants mentioned some difficulties; (1) shortcomings in the functional literacy of some populations, especially rural ones; (2) the lack of common use of SMS in Colombia as it is a resource frequently used by commercial marketers and (3) the fear that the sending of responses through this platform will generate costs for the respondent.

The participants suggested formats like social networks and popular chat platforms. However, they noted that for those who do not have them or know how to manage the technologies they would have difficulties to respond to these types of surveys.

Group participants preferred short questions in general, formulated in clear and simple language, that resort to concise and practical examples to facilitate their comprehension, with few answers and easy-to-remember options. Therefore, younger rural men openly expressed low disposition to participate in studies on habits related to alcohol or tobacco consumption or related health habits, because they feel they don’t lead to policies that favor their own health conditions.

When administering the survey during UGTs, participants agreed on duration as a critical factor to be considered in implementation. Most stated that surveys should only be 5 to 10 minutes for them not to lose attention. On this point, KIs and the younger urban women, younger rural men and rural adult women FGD pointed out the importance of openly indicating the survey’s duration and the number of questions. Additionally, the adult rural men UGT and KI interview expressed the importance of considering connectivity restrictions presented in some geographical areas in Colombia, because they consider it a limiting factor in some population sectors.

Here we have terrible cell phone signal, it almost never works. One has to call someone 20 times to be able to reach them, or to pace constantly to find a better signal. (Adult rural men UGT)

Participants did not show a consensus on the most appropriate time to administer the survey, preferences were associated with their lifestyle particularities. They clarified that the first tendency is that young people do not have any motivation to answer the calls. Younger rural men and adult urban men declared that ‘there is never a good time to answer surveys’. On the contrary, the adult rural men considered that any moment is appropriate to respond to surveys.

Finally, the rural adult men and rural adult women’s FGD, urban adult men’s FGD; and younger urban men’s UGT didn’t show any preference for a female or male voice for the survey; the other participants favored the female voice because they considered it more trustworthy and pleasant. While the rural adult women and urban adult women’s FGD preferred adult voices, the younger urban women’s FGD reported that they find younger voices more appealing. Despite these differences, all groups agreed with the fact that the voice should be firm and confident when formulating the questions; that it should inspire empathy and trust; sound natural and paused instead of ‘*robotic*’ and combine a steady volume with intonation changes to help the interviewee maintain attention and increase comfort during the call.

## Discussion

This study explored the perceptions, feasibility and potential strategies to increase the acceptability and response rate for NCD risk factor surveys administered through mobile phone technology, including IVR and SMS. A sequential exploratory qualitative design was employed and results are being used to enhance the design and implementation of these types of surveys in Colombia, as recommended by previous studies [2,4,22].

First, we found that phone calls from unknown numbers are not trusted and might be perceived as a medium to commit criminal or undesirable activities. Intensive information dissemination to generate trust in the survey through other media channels was frequently mentioned by participants including the provision of a contact number and website link to confirm the information provided, learn more about the study or even complain about the survey’s experience. Using additional media platforms (television, radio, newspapers) leading up the IVR launch or providing a contact number for the user was also proposed in several studies in Uganda aiming to adequately sensitize communities and mitigate distrust caused by phone calls from unknown numbers [3,11,12]. Other research has identified Colombia as one of the countries where more women than men have reported apprehension towards harassment and security issues (theft or fraud) as principal barriers to mobile phone ownership and usage. That report emphasized the need to ease these concerns that would otherwise further the gender gap in mobile phone ownership and usage by guaranteeing that credit can be refilled privately and remotely, launching awareness campaigns and via the creation and promotion of legal and policy frameworks that address mobile phone harassment [23].

Furthermore, our results show the importance of including precise information in the introduction on survey objectives, the institution responsible for it, the confidentiality of the data and the social contribution made by responding [24]. A study in Uganda that also conducted IVR surveys for NCD risk factors determined that some users expressed misunderstanding with the informed consent portion of the survey due to its content and short duration. That study proposed contacting the participants via SMS or a voice call beforehand and providing a contact number once they had decided to opt-in to answering the survey, which would allow participants more time to process key information related to the MPS [12]. It should also mention the mechanisms through which phone numbers were obtained [25].

In addition, the introduction should convey the message that this is an officially approved survey that is being implemented by a public health agency or nationally recognized research institution [25] and that making sure this information is available contributes significantly to improving the levels of confidence and commitment of the respondents [24].

Second, these results suggest that different population groups have variable survey preferences according to phone-related skill levels, age, availability and educational level. Several studies have analyzed how the ‘digital divide’ has become a challenge and barrier for some populations [5,24] especially the elderly because for some access to mobile technology does not necessarily imply advanced or even moderate technological capacity [26,27]. The lack of digital literacy can cause feelings of frustration and inability to use digital devices [28], which could be a limitation in completing the MPS. For example, adults and seniors with lower educational levels prefer the IVR format, while younger and more educated people feel more comfortable with SMS or other digital platforms (web applications). Thus, a well-designed health-related MPS should be fairly accessible to the general population if it takes elements used in the interfaces of other sectors (e.g., mobile banking, etc.). The results of this study suggest that considering a mixed approach with IVR and SMS for different population groups is appropriate.

Third, to increase response rates and minimize attrition, all participants recommend a short questionnaire of no more than 10 minutes, which coincides with the results of the pilot carried out by the World Bank in Peru and Honduras, but differs from early pilot testing of the IVR survey outside of Latin America like those made in the USA, where the suggested duration time ranges between 18 and 31 minutes [4].

Use of economic incentives to motivate participants to answer the survey has been suggested in previous studies [4,29]. Another study brought to light the potential ethical and data quality considerations associated with whether the participants received the economic incentive after completing the survey or if they received a partial credit independent of their survey completion status. On one hand, encouraging participants that they will receive a credit after finishing the survey could potentially yield inaccurate data throughout the survey question responses. On the other hand, it may be more reasonable to provide partial credit as the justifications for why the participant was unable to complete the survey are unknown; however, the latter approach may prove more costly and lower the response rate [30]. Our study, which offered the economic incentive upon completing the entire MPS, showed that perceptions on incentives in terms of airtime are appealing in general. However, some population groups prefer different types of incentives. In addition, when analyzing economic incentives, some participants stressed the importance of emphasizing the social contribution of participation, not just the monetary incentive.

Finally, our findings showed that the distribution of respondents to specific age groups and education levels might be skewed based on mobile phone use skills and coverage. A study that performed an MPS in La Paz, Bolivia on patients with NCD determined that older age adults, females and people with lower health literacy levels have less of a chance of owning a phone and thus limiting their effective participation and contribution towards mHealth initiatives. The previously mentioned study’s findings concluded that SMS interventions are viable when applied to that context and similar LMICs as the study demonstrated its overall ability to engage NCD patients including those with socioeconomic risk factors for poor health outcomes [31]. In the long term, this problem is likely to improve, but in the short term, more emphasis could be placed on monitoring trends than on finding population estimates.

## Limitations

This study has several limitations. First, our urban sample was composed of people from the city of Bogotá and the rural sample by people from nearby towns. Their views and perceptions may not be completely similar to other areas of Colombia. Second, the mobile telephone network coverage in Colombia is different across urban and rural areas, so it is necessary to extend the study to other areas of the country, especially those in which the signal is unreliable, in some cases, when the calls cut, it limits the application of the survey in UGT and this condition can negatively affect the sample, as shown by previous studies [32]. Finally, the KIs interviewed were a convenience sample that only included women, which can be a bias in the perception of the topics addressed in the study. Subsequent studies should also consider the opinion of male researchers and decision-makers.

## Conclusions

The possibility to obtain data through mobile phone technology represents an opportunity to improve monitoring of NCD prevalence and evaluation of interventions, particularly as a complement of face-to-face surveys. Automating data collection in such a way might improve not only the timeliness of data but also its access and legitimacy by increasing accountability and buy-in. Mobile phone technology has already shown the potential to be effective in managing NCD [12,30,31]. This study explores further and provides support for the potential role of mobile phone surveys as important tools to improve the information for public health policy decision-making across LMICs [33]. Studies such as the one described here are key to reconciling the rapid and expanding development and use of technology with the natural reluctance of some to assimilate such uses and the difficulty many may face in disentangling legitimate from illegitimate contacts.

## Paper context

The high costs and logistics of data collection for population-based face-to-face surveys often lead to long delays before survey statistics become available to policymakers, particularly in low- and middle-income countries. Mobile phone surveys (MPS) have the potential to fill these gaps. We explore the perceptions, feasibility and strategies to increase the acceptability of MPS for noncommunicable diseases using interactive voice response in Colombia. This study presents key steps to improve the acceptability of MPS.
